# Nontraditional Data in Pandemic Preparedness and Response: Identifying and Addressing First- and Last-Mile Challenges

**DOI:** 10.2196/85540

**Published:** 2026-04-29

**Authors:** Mattia Mazzoli, Irma Varela-Lasheras, Sónia Namorado, Constantino Pereira Caetano, Andreia Leite, Lisa Hermans, Niel Hens, Polen Türkmen, Kyriaki Kalimeri, Leo Ferres, Ciro Cattuto, Daniela Paolotti, Stefaan Verhulst

**Affiliations:** 1 ISI Foundation, Via Della Rocca 20, Turin, 10123, Italy, 39 011 6603090; 2 Department of Epidemiology, National Institute of Health Doctor Ricardo Jorge, Lisbon, Portugal; 3 NOVA National School of Public Health, Public Health Research Centre, Comprehensive Health Research Center, Associated Laboratory in Translation and Innovation Towards Global Health, Clinical Academic Center, NOVA University Lisbon, Lisbon, Portugal; 4 Interuniversity Institute of Biostatistics and Statistical Bioinformatics, Data Science Institute, Hasselt University, Hasselt, Belgium; 5 Centre for Health Economics Research and Modelling Infectious Diseases, Vaccine and Infectious Disease Institute, University of Antwerp, Antwerp, Belgium; 6 The Data Tank, Brussels, Belgium; 7 Data Science Institute, Universidad del Desarrollo, Santiago de Chile, Chile; 8 The GovLab, New York University, New York, NY, USA; 9 Interuniversity MicroElectronics Center, Studies in Media, Innovation and Technology, Vrije Universiteit Brussels, Brussels, Belgium

**Keywords:** nontraditional data, pandemic preparedness, pandemic response, data science, epidemic modeling

## Abstract

The COVID-19 pandemic served as an important test case of complementing traditional public health data with nontraditional data, such as mobility traces, social media activity, and wearable data, to inform real-time decision-making. Drawing on an expert workshop and a targeted survey of epidemic modelers in Europe, this study assesses the promise and the persistent limitations of such data in pandemic preparedness and response. We distinguish between “first-mile” challenges (obstacles to accessing and harmonizing data) and “last-mile” challenges (difficulties in translating insights into actionable policy interventions). The expert workshop, convened in March 2024 in Brussels, brought together 50 participants, including public health professionals, data scientists, policymakers, and industry leaders, to reflect on lessons learned and define strategies for better integration of nontraditional data into epidemic modeling and policymaking. The accompanying survey, gathering experiences from 29 modelers, offers empirical evidence of the barriers faced by modelers during the COVID-19 pandemic and highlights areas where key data were unavailable or underused. The experiences collected through the survey and workshop resulted in ten key actions and three overarching recommendations for public entities, data providers, and stakeholders. Our findings reveal ongoing issues with data access, quality, and interoperability, as well as institutional and cognitive barriers to evidence-based decision-making. Approximately 66% of all datasets had at least one access problem, with data sharing reluctance for nontraditional sources being double that of traditional data (30% vs 15%). Only 10% of respondents reported that they could use all the data they needed. These limitations included issues related to timeliness and granularity of data, as well as issues with linkage, comparability, and biases. To overcome these hurdles, we propose a set of enabling mechanisms, including data inventories, standardization protocols, simulation exercises, data stewardship roles, and data collaboratives. For first-mile challenges, solutions focus on technical and legal frameworks for data access. For last-mile challenges, we recommend fusion centers, decision accelerator laboratories, and networks of scientific ambassadors to bridge the gap between analysis and action. We argue that realizing the full value of nontraditional data requires a sustained investment in institutional readiness, cross-sectoral collaboration, and a shift toward a culture of data solidarity. Grounded in the lessons of the COVID-19 pandemic, the study can be used to design a roadmap for using nontraditional data to confront a broader array of public health emergencies, from climate shocks to humanitarian crises.

## Introduction

### Background

Throughout the COVID-19 pandemic, decision-makers around the world looked for timely and quality information to inform their response efforts. Yet, much of the data traditionally used for public health (eg, public health–based surveillance data, health care–based data, and clinical trials) were not available fast enough or at the scale or the coverage needed for a crisis that extended beyond national borders and required a multisector, timely response. As a result, many in the scientific community turned to new, nontraditional data sources and cross-sectoral collaborations to fill these gaps and accelerate knowledge generation to support decision-making. We acknowledge the importance of mathematical, statistical, and artificial intelligence tools to interpret data and provide support for evidence-based policymaking. However, here, we explicitly focus on the two ends of the process, that is, the availability and retrieval of critical data and their power to inform public health decisions.

Nontraditional data (NTD) is often defined as “repurposed data that can be digitally captured (eg, mobile phone and financial data), mediated (eg, social media and digital traces), or observed (eg, satellite imagery)” [1-3], often using new and privately held technology [3]. As such, it is an umbrella term for information not originally collected for public health but repurposed for that purpose (Table 1). As such, feeds are often generated continuously and at a population scale, they offer more granular and more real-time data, helping public health responders, governments, and civil society organizations to respond more effectively.

**Table 1.** Properties of traditional and nontraditional data (NTD).

	Traditional data	NTD
Primary intended use	Population health	Business optimization, transport planning, environmental control, algorithm optimization, social behavior analysis, and flu-like illness monitoring
Collection method	Analog and digital	Digital
Coverage type	Groups targeted through recruitment, health care seeking, and notifiable diseases	Groups defined by digital service use and technology adoption - often scalable to the population level
Structure	Structured	Often unstructured
Most common biases	Reporting delays, underreporting, unequal health care–seeking behavior, geo coverage, testing capacity, representativity, unequal health access, heterogeneous reporting standards, and noise	Costly, infrequent sampling, recall bias, self-selection bias, representativity, digital divide, data ownership, low granularity, weak link to contact patterns, unstructured, demographic bias, privacy and interoperability, infrastructure sustainability, incomplete metadata, and heterogeneous quality
Example	Cohort studies, clinical trials, traditional contact tracing, consultation rates, and notifiable diseases	XDR^a^ mobility data, participatory surveillance, social media data, and digital contact tracing

a XDR: extended detail record.

The potential of NTD to inform the response to public health emergencies has already long been recognized. For example, during the 2014 to 2016 West Africa Ebola outbreak, anonymized mobile phone call records were used to map population movements and shape travel restriction policies in Sierra Leone [4]. Similarly, Google search queries have been used to predict and offer early warnings on diseases, including Zika [5]. The use of NTD has been an important aspect of many of these efforts. Its use was further highlighted during the COVID-19 crisis, illustrating how such a rapid and granular data source can be used to inform the response to a public health crisis.

**Figure 1.**
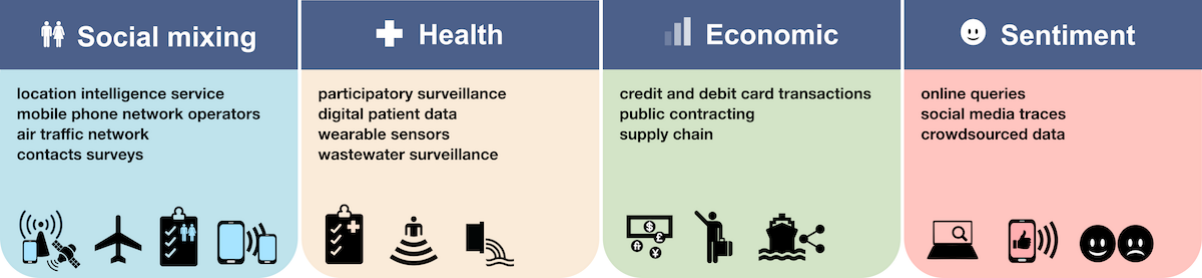
Nontraditional data types and sources. Schematic representation of the 4 types of nontraditional data and a sample of the main respective sources. Readers may refer to the text for a more extensive description of data sources and their use.

Typical NTD sources include aggregated and anonymized mobile location traces, information on retail purchases, social media posts, and data sourced from wearable devices, for instance, on heart rate or temperature [3]. NTDs have been classified into 4 main types: health, social mixing, economic, and sentiment data (Figure 1). Applications to pandemic response are exemplified below.

### Health

At the core of pandemic response stands the monitoring of population health. At the beginning of the pandemic, researchers quickly repurposed preexisting crowdsourcing platforms to monitor COVID-19 circulation and related behaviors [6,7]. The participatory surveillance [8] platform InfluenzaNet [9] has collected flu-like symptoms and health-related behaviors from volunteers across Europe since 2003 to analyze country-level incidence trends on a weekly basis [10]. While volunteers report symptoms to their respective country’s unique platform, all national platforms within the InfluenzaNet umbrella adopt standardized approaches to reporting, facilitating cross-country analysis. InfluenzaNet expanded its metrics infrastructure in 2020 to include COVID-19 data. Similar efforts are found in the United States with OutbreaksNearMe [11], in South Africa with CoughWatch [12], and in Australia and New Zealand with Flutracking [13]. In many cases, symptom reports are collected through mobile apps, as in the case of a mobile app [14] launched by King’s College London and ZOE, a science and nutrition company. These data helped public health officials in the United Kingdom to identify early indicators of illness in the emergent stages of the pandemic.

Smart thermometers and other wearable sensors were a valuable data source for analyzing trends in fever in the population. Wearable sensor data [15] collected by Kinsa’s network of smart thermometers [16] have been used to analyze fever patterns and develop outbreak forecasting maps across several countries. Thermometer data were already used in 2012 to forecast influenza outbreaks and were repurposed for COVID-19 during the onset of the pandemic. A wide range of stakeholders used these data, including pharmacies, schools, and other decision-makers. In Germany, wearable technology data collected by Thryve, in partnership with the Robert Koch Institute, created a data donation platform [17] for improved fever detection research in the spring of 2020. Among health-related repurposed data are news media records. While being focused on informing the general public of ongoing health threats, global parsers and visualizers, such as HealthMap, help collect and monitor news of disease outbreaks worldwide [18]. Using trusted social and news media, recent efforts have built open-source global datasets and mappers for spatiotemporal health threats surveillance [19,20]. At finer scales, wastewater-based epidemiology, which is based on sampling of sewage waters, proved to be a powerful tool during the pandemic to achieve a more granular and unbiased method to monitor disease circulation [21,22]. Wastewater-based epidemiology can detect viral traces of SARS-CoV-2 among other biological and environmental contaminants and reflect transmission dynamics in untested communities [23], allowing for earlier detection of outbreaks. In Hong Kong, wastewater surveillance efforts found the first evidence of the spread of the Delta variant and informed public health interventions [24].

### Social Mixing

On a geographic scale, mobility patterns encode the mixing of residents of different locations, allowing modelers to estimate the spatial spread of the disease and suggest spatially targeted interventions. At the population level, contact data encode interactions at risk of contagion, allowing modelers to study disease transmission across demographic groups and suggest setting-specific interventions. Mobility records were published regularly after the early days of the pandemic through *Data for Good* [25] programs at diverse spatial scales and temporal resolutions. Google, for instance, regularly published human mobility data through its Community Mobility Reports [26]. These data regarded worldwide regions and included trends around commonly visited location types (eg, retail, workplace, and residential areas) that, when combined with census data, helped inform epidemic modeling to support decision-makers. Social media companies’ *Data for Good* programs, such as Meta [27] and Cuebiq [28], assisted epidemic modelers in providing critical insights for public health measures and population studies. Telecommunications companies [29] played an important role in tackling the pandemic in their respective countries, helping modelers and public health practitioners monitor and understand the heterogeneous population response to restrictions, as in the case of Telefonica Chile [30] and Orange France [31]. These data helped decision-makers assess the effectiveness of their policies, monitor population compliance, and tailor the public health responses. Beyond mobility patterns, social mixing data were typically collected from traditional contact surveys and diaries, which did not scale well at the national level and did not offer longitudinal or real-time follow-up of human behavior. Following prepandemic efforts such as POLYMOD [32], online contact surveys reemerged in the pandemic’s early days thanks to new crowdsourcing tools at the European level, such as CoMix [33], an initiative funded by the European Commission (EC) that quickly gathered public mixing and sentiment data during the COVID-19 pandemic. The weekly survey started in March 2020 in Belgium, the Netherlands, and the United Kingdom before expanding to 17 additional countries across Europe. Marketing firm Ipsos MORI recruited participants via social media advertisements and email campaigns to crowdsource reported social contacts, assisting with national policy evaluation.

### Economic

The pandemic shifted consumers’ needs and habits, impacting local businesses’ revenues and opening new market opportunities. Economic NTD helped policymakers monitor the impact of the pandemic on local businesses and the population’s spending habits. Data on electronic transactions [34] provide information on the time, location, and purpose of people’s purchases, proxying their economic wealth. Electronic transaction data from credit cards were used to measure the reduction in revenue [35] of economic activities during the COVID-19 pandemic.

Supply chain data allow monitoring the status of the production and distribution of goods, providing logistics organizations with important insights to optimize the goods pipeline. Using maritime traffic data collected via a global network of Automatic Identification System receivers, the Marine Traffic Research Lab studied the impact of the pandemic on the shipping industry [36]. Open contracting records refer to records of public contracts from administrations. They are used in many countries to increase transparency and combat corruption in public spending. In Ecuador, the National Public Procurement Service launched a public-facing dashboard of emergency procurement contracts during the COVID-19 pandemic [37].

### Sentiment

While aiming at controlling disease circulation, interventions had an impact on population mental distress and were partially received by citizens, giving rise to misinformation exposure and loss of adherence to policies over time. Sentiment NTD allowed researchers and organizations to monitor people’s perceptions, mental health, and exposure to misinformation. Online search queries and social media data were valuable tools to understand the impact of online behavior on the pandemic progression and monitor the population’s perception of restrictions. Google search trends and tweets were used by German researchers to create a future-oriented early alert system [38] that could be used to build phenomenological and mechanistic models to forecast cases and hospitalization trends. Tweets before and during the pandemic were analyzed by researchers in Italy to analyze the potential health threat driven by online misinformation [39] related to the pandemic. The same data were used by researchers in the United States to integrate public health data with social media data, developing an early alert system for outbreak detection [40]. However, sentiment data do not stop at online discourse on social media; the worldwide COVID-19 trends and impact survey [41] conducted by Meta collected important information, among others, on the population’s mental health. Over the course of the pandemic, respondents transmitted self-reported feelings of anxiety and depression, for the first time collecting important insights on the population’s mental distress and risk perception during a pandemic. While CoMix [33] collected both self-reported behavioral data and sentiment from participants, other efforts aimed to incorporate a wider variety of data streams related to people’s perception of the pandemic. For example, 6 European public health agencies collaborated during the pandemic to create PANDEM-Source [42], an IT surveillance tool that integrates both traditional case counts and nontraditional social media reports for pandemic surveillance. Results feed into PANDEM-2 [43], a real-time data dashboard for decision-makers and health experts.

NTD can help fill critical gaps in traditional data during public health emergencies by increasing the speed and volume of data collection. When combined with traditional data, NTD can provide a more comprehensive understanding of the problem and support rapid decision-making. However, alongside the promise, COVID-19 also revealed challenges involved in the use of both traditional data and NTD. The early months of the COVID-19 pandemic were characterized by ad hoc collaborations, which revolved around the use of NTD sources: mobility dashboards built overnight [44], wastewater pilot studies [24], and hastily negotiated data sharing agreements [45].

A noteworthy example of ad hoc collaboration between public bodies and the private sector was the EC’s collaboration with European Mobile Network Operators (MNOs) [46]. Within just a few months, 17 MNOs across 22 EU states and Norway provided access to mobile phone data with the aim of understanding the geographical spread of the disease and assessing pandemic interventions. In addition to European-level initiatives, many subnational regions, such as the Valencian region of Spain [47], combined data from mobile operators with data from Facebook, Google, and government ministries to evaluate the impact of mobility restrictions on COVID-19 spread. Challenges that emerged from these pioneering initiatives included data harmonization and privacy guardrails.

Even after the pandemic, we can find virtuous examples of public-private collaborations allowing NTD collection aimed at making data publicly accessible for research. One institutional example is represented by the initiative of the Transport Ministry of Spain [48], regularly making publicly available origin-destination matrices [49] of mobility flows at high temporal and spatial resolution in Spain since early 2020. This ongoing initiative is a collaboration between the Ministry, Orange Spain, and Nommon and provides a curated and poststratified, ready-to-use dataset of human mobility stratified by multiple sociodemographics. On a more bottom-up side, the community of researchers is also responding to the growing lack of NTD accessibility. This is an example of the R2M2P2 Consortium: Readying Regional Mobility data for Modeling Pandemic Preparedness [50], led by the Erasmus Medical Center in collaboration with Dutch universities and the national public health agency RIVM. The aim of this effort is to identify useful mobility data sources and set up a pipeline providing usable and accessible mobility data to inform transmission models to respond to key public health questions on the spread of respiratory infections in the Dutch population.

These innovations helped highlight how diverse data streams, when responsibly harnessed, can quicken detection times and potentially reduce mortality and morbidity. These experiences served as an important test case for the integration of these new sets of data into epidemic modeling and decision-making for pandemic response. However, they also exposed deep structural weaknesses, such as fragmented governance, limited interoperability, and a chronic skills gap. Questions remain around how these data can best be used in a pandemic crisis, namely, regarding their limitations and the investment needed to replicate efforts from COVID-19 in future public health emergencies. A better understanding of these challenges may provide insights and lessons for future efforts to combat public health emergencies.

Traditional data exhibit known biases (eg, as reporting delay, geographical coverage, and health care accessibility disparities among others) that modelers typically take into account using Bayesian inference, sample poststratification, and artificial intelligence–based synthetic data generation techniques. However, NTDs are not exempt from biases and have their own limitations, most commonly being sample bias resulting from technology adoption divide, data generation noise due to self-reporting bias and unequal representativity among population groups, unequal spatiotemporal coverage, and poor granularity. In Table 1, we summarize the most common biases for both data types. Modelers adopt similar techniques to those used for traditional data to account for NTD biases, but effective approaches are case specific and data specific. Beyond the focus of the survey and workshop on the challenges of accessing and integrating NTD into evidence-based policy, recent studies have mapped and addressed thoroughly known biases of NTD [2,51-53].

This study is based on the structured discussions held during an expert workshop on the use of NTD to counteract pandemics conducted in March 2024 in Brussels, along with the results of a survey on data readiness and availability disseminated among the European modeling community conducted the year before. The workshop convened public health professionals, data scientists, policymakers, industry leaders, and other stakeholders to reflect on lessons learned and define strategies for better integration of NTD into epidemic modeling and policymaking. The accompanying survey offers empirical evidence of the barriers faced by modelers during the COVID-19 pandemic and highlights areas where key data were unavailable or underused. Together, these 2 sources form the basis for 10 key lessons learned and proposed actions about the role of NTD in pandemic preparedness and response, which we outline below.

On the basis of the experts’ opinions and the conducted survey, we draw a set of recommendations that establish a framework for the use of NTD in ongoing and future public health and other crises. From the survey responses, 2 main categories of obstacles emerged: “first-mile” and “last-mile” challenges. First-mile challenges refer to the difficulties of rapidly identifying and accessing relevant data when a crisis begins; these include legal constraints, unclear stewardship, and difficulty in the application of the FAIR [54] principles, that is, lack of interoperability and, critically, unavailability of data. Last-mile challenges are more nuanced and often less recognized but are nonetheless critical. They concern the difficulty of translating complex data into actionable insights that decision-makers can understand, trust, and apply. Together, these challenges represent a formidable and persistent obstacle to the use of NTD in public health emergencies. To move beyond improvisation during a crisis, we argue for a more proactive, institutionalized, and scalable approach to using NTD streams before, during, and after public health emergencies.

## Survey on Data Availability and Data Needs

In 2024, we conducted a survey [55] to better understand data availability, use, and unmet needs during the COVID-19 pandemic, primarily in the European modeling community. Specifically, the survey aimed to gather information about which types of data respondents drew on during the COVID-19, obstacles and challenges they faced, and whether and how these data were used to shape policy.

The survey was divided into 3 sections. The first section aimed to collect the research questions and modeling approaches used by researchers participating in the survey. The second section aimed to identify (1) what data were available and were used during the pandemic, (2) how data were accessed and what problems were encountered in accessing the data, and (3) what quality problems were encountered when using the data. Finally, the third section aimed to understand what data were needed but not used by these research groups and whether these unmet needs were the result of data unavailability or the result of data quality and access issues. Throughout the survey, the data were categorized into traditional epidemiological data (eg, tests, hospitalizations, deaths, and vaccination) and nontraditional epidemiological data, that is, data types not collected with epidemiological purposes and/or relatively new data types (eg, mobility, contacts, and wastewater data), so that in addition to each individual data type, we can have a broader view on traditional versus NTD. Overall, the survey was composed of 11 questions, of which 10 were closed-ended questions, with the last open question regarding the purpose of the data use.

Using a purposive sampling approach, we circulated the survey through the ECDC Modeling Hubs mailing list, a community populated by many public health emergency responders during the COVID-19 pandemic and the ESCAPE consortium. The aim was to target as many epidemic modelers as possible in Europe. In total, 29 experts (individuals and groups) from academia, government agencies, and the private sector responded to the survey.

## Pandemic Response as a Hybrid Enterprise

In this section, we present the results obtained from our surveyed sample of the modeling community in Europe. All reported statistics are purely descriptive and reflect the responses of our sample; as such, we do not infer statistics or generalize to broader populations.

**Figure 2.**
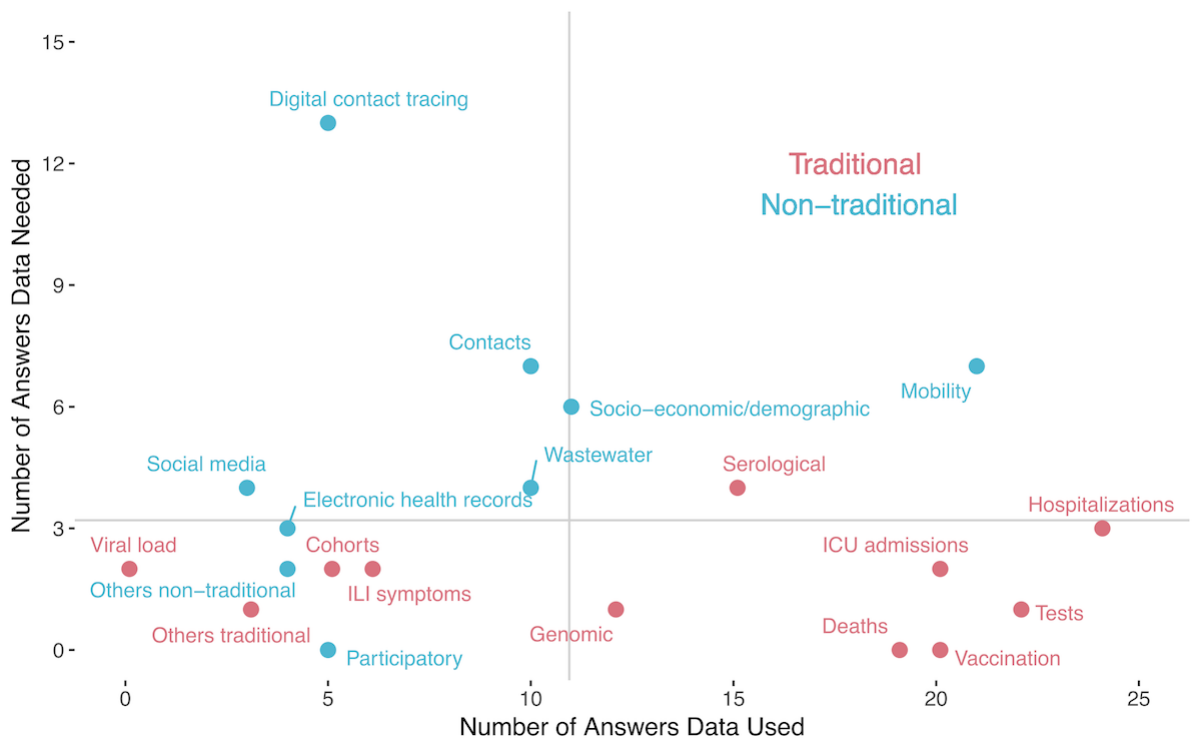
Comparison of the use (x-axis) and the need (y-axis) of the different types of data reported by respondents. Vertical and horizontal gray lines represent the mean number of answers for all data types used and needed, respectively. The upper-right quadrant represents data highly used and needed; the upper-left quadrant represents data highly needed but not highly used; the bottom-right quadrant represents data highly used but not highly needed; and the bottom-left quadrant represents data neither highly used nor highly needed. Reported values are the result of closed-ended multiple-choice questions. ICU: intensive care unit; ILI: influenza-like illness.

The survey responses suggest that NTD sources have moved from fringe to mainstream, indicating that pandemic modeling and response among participant modelers have in many ways become a hybrid enterprise, in which NTD often complement and augment more traditional (if somewhat slower) sources. For instance, 90% of enquired modelers reported using at least one nontraditional stream alongside more conventional sources. Among nontraditional data types, mobility was dominant (72% of respondents), alongside other nontraditional sources such as socioeconomic and demographic, contact, and wastewater data (Figure 2). An equally striking aspect of the survey finding was the direct relevance of such data to inform the response to the pandemic. Seventy-nine percent of the respondents stated that they used data to inform public health policies, with the largest number of these (83%) stating that they used mathematical models to translate data into insights used to inform policymaking, followed by statistical models (48% of respondents). More specifically (and in addition to recent work conducted by the GovLab on identifying use cases for the use of NTD [56]), respondents reported their need for NTD to achieve the following goals:

- monitoring adaptive behavior to interventions across sociodemographic groups
- assessing the age-specific impact of interventions on disease transmission
- understanding the role of socioeconomic factors in disease spread
- better informing and validating models assumptions
- better calibrating complex models, particularly behavioral components
- improving short-term predictions at regional and local scales
- assessing the risk of health care system saturation
- better tracking the spatial invasion patterns of disease
- identifying the most infected areas and high-risk population groups with finer higher granularity
- tracing transmission chains and assessing estimating the distributions of secondary cases
- broadening and sharpening the scope of epidemiological studies

Moreover, NTDs were needed to assess the risk of health care system saturation, better track the spatial invasion of disease, assess most infected areas and population groups with higher granularity, trace transmission chains, and assess distribution of secondary cases.

## Workshop on First- and Last-Mile Challenges for Data Needs

On March 22, 2024, we held a workshop at the University Foundation in Brussels to better understand the role (including limitations) of NTD. Attendance at the Workshop was upon invitation. We assembled a high-level expert panel reflecting the workshop’s interdisciplinary goals, bringing together approximately 50 participants from diverse sectors:

- Private sector representatives from industries, such as telecom companies, which typically own and provide relevant NTD
- Academic sector leading European scientists and project partners who played pivotal roles during the pandemic response
- Public health representative officials from national public health institutes (eg, Portugal and Switzerland) and European institutions (eg, HERA)

**Figure 3.**
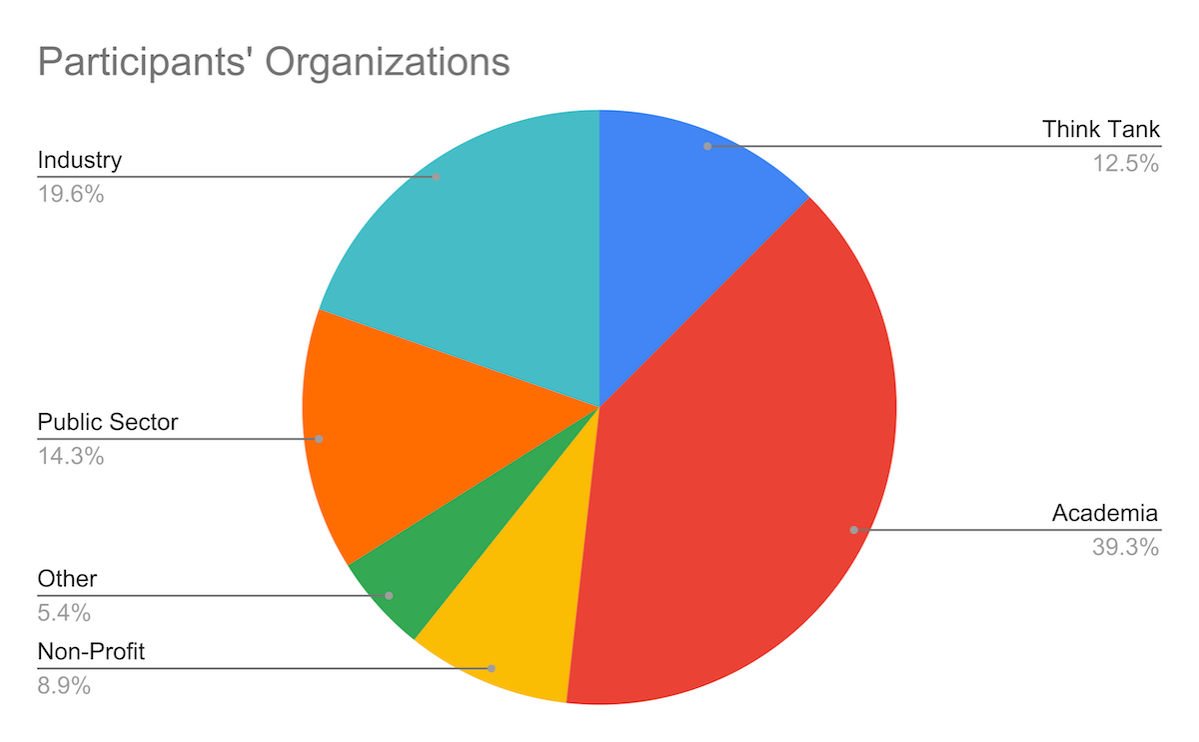
Workshop participating organizations. Pie chart representing the sector of the 50 participants in the workshop. See the Acknowledgments section for the full list of participants and affiliations.

Attendees were selected based on their expertise and background. Figure 3 shows the provenance of the invited participants.

The workshop’s specific objectives included the following:

- Advancing the dialogue on leveraging NTD for pandemic preparedness
- Drawing insights from experts and practitioners in relevant fields
- Building meaningful collaborations to enhance pandemic response efforts
- Co-creating high-level recommendations and a roadmap for European decision-makers

Discussions and activities were structured around 2 main themes: “Data for Preparedness” and “Data for Pandemic Response.” Participants were divided into working groups to explore these topics through 3 key sessions.

1. First-mile challenges: addressing readiness to access NTD in case of a pandemic. Questions included were as follows: Are we prepared to access and reuse NTD for preparedness and response? What challenges remain from the previous pandemic?
2. Prototyping first-mile solutions: This session focused on refining and defining challenges, gathering quick feedback, identifying areas with the greatest impact or feasibility, and proposing concrete actions and recommendations.
3. Last-mile challenges: Exploring how to ensure data insights are translated into actionable decision-making. Discussions centered on identifying key decision-makers, facilitating partnerships between data, scientific, and policy communities, and fostering effective communication.

We adopted this established framework [57-61] as an organizing principle for the workshop. In pandemic response, the “first mile” refers to the ability to quickly obtain both traditional data and NTD and make them available for reuse. The “last mile” focuses on converting that data-driven knowledge into actionable insights and ensuring it is effectively applied by policymakers and public health authorities.

The final plenary session united all participants to review and discuss the solutions proposed during the previous sessions. Discussions in all sessions were reported by in-person note takers.

## Difficulties and Challenges Reported by Participants to the Workshop and Respondents to the Survey

### Overview

The pandemic revealed some significant challenges that remain unsolved. Both the convened experts and the survey aimed to better understand the nature of these challenges and how they might be overcome to do better in future health emergencies.

**Figure 4.**
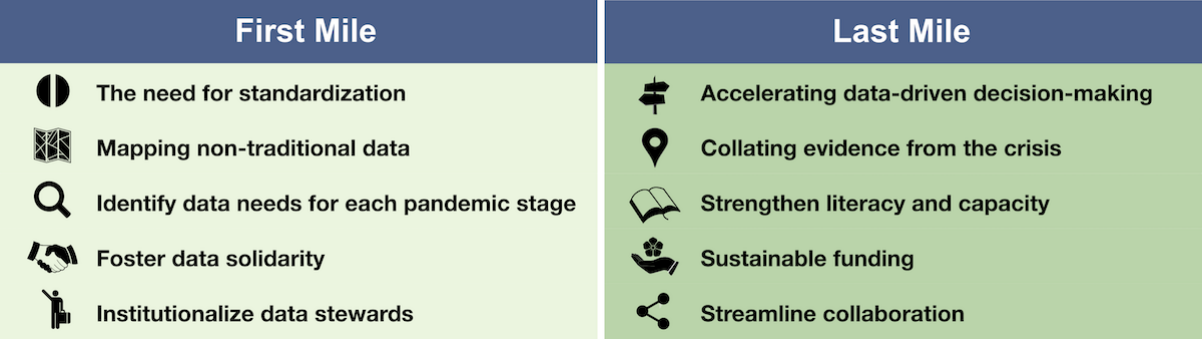
Proposed actions addressing first- and last-mile challenges. Schematic figure illustrating the 10 proposed actions summarized within the first- and last-mile framework.

One of the overarching findings of the survey is that the use of data [62] to inform the response to COVID-19 and other public health emergencies can be broken down into 2 main challenges: access, in which data are identified, sourced, and processed; and translation, in which data are used for policymaking and meaningful interventions. We call these first-mile and last-mile bottlenecks (see Figure 4 for a summary of the two). We used the same division to shape the structure of the workshop sessions, as this is a relevant and useful framework to analyze the bottlenecks of data pipelines in pandemic response. First-mile bottlenecks often get the lion’s share of attention; however, last-mile shortcomings are equally significant in the way they impede the conversion of insights into timely action. In other words, access without translation is potentially as limiting as a simple lack of access.

### “First-Mile” challenges

First-mile hurdles can arise at any stage of a public health crisis. They refer to difficulties responders face in identifying and accessing relevant data streams [63]. Obstacles can include opaque ownership and licensing, a lack of existing data sharing agreements, missing metadata, and quality issues. Technical fragmentation—including nonstandard formats and incompatible APIs—is another impediment. Two key obstacles highlighted by workshop participants and survey respondents included a lack of standardization and a shortage of incentives for sharing, leading to what the experts labeled reduced “data solidarity” [64].

The survey quantifies some of these pain points. According to experts, approximately 66% of all datasets used by respondents—traditional and nontraditional—had at least one access problem (S1 Fig in Multimedia Appendix 1). The proportion of issues linked to data sharing reluctance in the context of NTD was double that of traditional data (30% vs 15%; see S1 Fig in Multimedia Appendix 1). Furthermore, only 55% of nontraditional streams were publicly available or obtainable via government agreements, compared to more than 83% for traditional data (S2 Fig in Multimedia Appendix 1). In addition, data quality problems were also prevalent. Despite being rare, preparation and processing issues existed across both traditional data and NTD (12% vs 10%; S3 Fig in Multimedia Appendix 1). Only 4% of the datasets that participants reported (both traditional and nontraditional) had no quality problems; perhaps somewhat surprisingly, machine readability [65] issues were far more prevalent with traditional data compared to NTD (11% vs 2%). These quality issues may stem from the fact that individuals and organizations are less familiar with NTD requirements than traditional data. As most *Data for Good* programs were ceased after the pandemic, a further obstacle is found in data impermanence, which hinders data reusability and makes data pipelines more fragile in the face of new threats.

### “Last-Mile” Challenges

Last-mile issues surface at the opposite end of the data-to-policy pipeline. They hamper the process of turning analysis into actionable intelligence and meaningful interventions. Once again, there are multiple drivers of these limitations, including gaps in data and technical literacy among officials, weak institutional bridges between scientists and policymakers, and slow feedback loops. Mismatches between the types of data available and requirements for policymaking can also play a role. As we have elsewhere argued [66], taking a question-based approach to evidence-based interventions can help align data with the most pressing and relevant policy issues.

At a broad level, the survey suggests a degree of success on this front. As noted earlier, 79% of respondents stated that their analyses fed directly into public health policy. Nonetheless, a further examination of the survey results suggests a number of obstacles that prevented data from actually being usable (S4 Fig in Multimedia Appendix 1). Besides data availability, these include limitations in the timeliness and granularity of data, issues with linkage [67], comparability, and biases. Only 10% of respondents reported that they could use all the data they needed. This was particularly relevant for NTD (eg, contact tracing [68], mobility, and contact data) and reflects a broader problem with data availability, which remains a significant barrier for data usage in policy making (S4 Fig in Multimedia Appendix 1).

## Ten Lessons Learned and 10 Proposed Actions

In this section, we outline key proposed actions in response to 10 lessons learned that emerged from the workshop and survey addressing first- and last-mile challenges.

### The Need for Standardization

Participants at the workshop stressed how the experience of reusing NTD during the COVID-19 pandemic revealed widespread inconsistencies in data formats, metadata, and quality metrics. These issues made it difficult to integrate and analyze data across sources and impeded timely insights. The expert workshop emphasized the urgent need to establish common standards not only for the data itself but also for metadata structures, reproducibility, and quality indicators. In particular, the lack of standardized approaches [69,70] to processing origin-destination matrices [71] in mobility data emerged as a significant obstacle for modeling [72] and understanding epidemics [73]. One suggestion was the establishment of a multisectoral task force to create a common set of questions and methodological standards, enabling smoother collaboration before the next crisis.

One virtuous example is the effort carried out through the MNOdata4OS and Multi-MNO project [74]. This initiative, developed by the European Statistical System [75], aims to create a consistent methodology for processing MNO data for official statistics throughout Europe. The project also includes defined quality standards and an open-source reference implementation of the outlined methodology. Notably, this ongoing work seeks to transition MNO data usage from Experimental Statistics [76] to official statistics by addressing challenges of data privacy, creating consistent legislation for long-term data access partnerships, and implementing standardized methodologies.

### Accelerating Evidence-Based Decision-Making

Advanced analytics mean little if they do not feed into policy; as we have seen, the last-mile challenges remain significant. The workshop, therefore, endorsed three “bridges” that could help advance evidence-based policy: (1) fusion centers [77], to merge and make actionable data streams; (2) decision accelerator laboratories [78], to prototype interventions and iterate on policy; and (3) a network of scientific ambassadors trained to translate data insights into clear-language policy and more generally to bridge science and practice. Together, these mechanisms can help move from insight to action more quickly. The group also recommended building a cross-border European community of epidemic modelers to expand access to evidence and ensure that no region is left behind in future emergencies.

### Mapping NTD

Discussion at the workshop highlighted how during the COVID-19 pandemic, as with prior and subsequent public health emergencies, many stakeholders were often not aware of NTD. Participants stressed this as a critical barrier and emphasized the need for a “data map,” that is, a comprehensive data inventory describing what datasets exist and rating them for accessibility, relevance, and quality. They also underlined the need for regular audits and harmonized metadata templates so that such an inventory would be searchable and valuable across a range of use cases.

The EU Health Data Space (EHDS) [79], currently collecting health data only, should begin to absorb such demand by building common standards, practices, and governance for health data reuse in research, innovation, and policymaking, integrating NTDs that are essential for fast public health response to health crises. Adopted in January 2025, the EU Health Data Space proposes HealthData@EU [80], an ecosystem of national platforms that establishes standards for data quality and use and facilitates the annual publication of data catalogs to find and use common data types across borders.

### Collating Evidence From the Crisis

Participants at the workshop stressed that while hundreds of initiatives during the COVID-19 pandemic used mobility, payments, or social media signals [3], lessons learned remain scattered and difficult to generalize.

The workshop highlighted the need for more research on how NTD is used, during which phases, and with what impact. Such a repository would include not only project descriptions but also tags, pipeline schemes, and metadata that allow sorting by disease stage, data type, and outcome at the level of intervention effectiveness based on these data. In this way, future public health professionals and researchers could quickly identify what worked, avoid redundancy, and inform fast and effective interventions while avoiding previous mistakes.

### Identify Data Needs for Each Pandemic Stage

We learned how identifying and aligning data needs with evolving goals at different stages of a pandemic is essential. Workshop participants highlighted the value of tabletop simulation exercises as a practical tool to explore evolving data requirements. The panel of experts suggested tabletop simulations to provide simulated environments [42] to explore hypothetical pandemic scenarios, revealing dynamic data requirements at various stages of public health emergencies. In doing so, they help policymakers and other stakeholders assess preparedness and response strategies, as well as identify critical data needs and data streams ahead of time.

### Strengthen Literacy and Capacity

We have noted a number of last-mile hurdles that limit policymakers’ capacity to transform NTD into actionable insights. In addition to those already listed, the challenges also include a significant skills gap among public health officials and other stakeholders. To address this, the workshop expert panel called for investments in both analytical training (eg, interpreting mobility or social media data) and “question literacy” [66]. Question literacy is an essential but underappreciated skill. It aligns data supply with data needs and can help prioritize scarce public resources and policymaker bandwidth (eg, by minimizing time spent searching for or analyzing unhelpful or unnecessary data).

### Foster Data Solidarity

A recurring theme of the workshop was the need to foster a culture of “data solidarity,” that is, the idea that data holders, especially in the private sector, should commit to sharing data responsibly and proactively during public health emergencies. This requires not only ethical and legal frameworks but also the cultivation of mutual trust and shared purpose among stakeholders and across sectors. Practical manifestations of data solidarity could include prenegotiated commitment frameworks, increased transparency around how data will be used, and the development of social licenses [81] through public engagement. In addition, data collaboratives [82], that is, expert groups based on public-private collaborations exchanging data to help solve public problems, and similar bodies have proven effective in promoting trust and data sharing. Across all these mechanisms, it is essential to establish incentives for sharing; these could include naming and framing schemes and access to aggregated insights for strong sharers.

### Institutionalize Data Stewards

The expert panel stressed that even when the incentives and structures for data solidarity exist, coordinating actual data sharing is a complex task, especially in the midst of a public health emergency. The workshop suggested the creation of dedicated “data stewards” [83] to help institutionalize and facilitate this process. Data stewards are individuals or groups responsible for building partnerships, conducting audits and risk assessments, facilitating internal coordination, and communicating externally with stakeholders. Their role has proven especially effective in nurturing and sustaining data collaboratives [82]. By making this a formal, resourced role, organizations can ensure they are better prepared to access and share data when a crisis occurs.

### Sustainable Funding

Long-term, sustainable funding and investment are critical to contrasting data impermanence, promoting data sharing and effective use of NTD. One of the clearest lessons from the COVID-19 crisis is that ad hoc or one-off funding mechanisms are inadequate and that long-term capacity requires sustained investment in infrastructure, human capital, and technology. Toward this end, the expert group recommended establishing a dedicated data fund, that is, public-private partnerships supporting data availability for evidence-based policies. The fund should be governed transparently, include mechanisms for rapid-response grants, and build in accountability through independent oversight.

### Streamline Collaboration Through Advanced Technologies

The expert group highlighted how policy, norms, and society are vital parts of any framework to promote the use of NTD. However, it should not be overlooked that advanced technologies themselves can play a transformative role, especially in encouraging more data sharing. Participants suggested considering technologies such as privacy-enhancing tools [84], Edge Computing [85], Data Sandboxes [86], Data Pods [87], and synthetic data [88], including social contacts [89] and mobility [71], beyond electronic health records. Many of these underlie, the emerging concept of “data spaces”—structured, rule-based environments in which data holders retain control over how their data are shared and used [90]. By investing in these technologies, governments and public health institutions can significantly strengthen their ability to collaborate while still maintaining public trust and protecting privacy and other rights.

## Conclusions

### Overview

The 10 lessons outlined earlier, as well as the survey results, point to a set of broader priorities for policymaking. In this final section, we distill the experience of COVID-19 into 3 overarching recommendations that can inform not only future responses to public emergencies but also steps that governments and other stakeholders need to take today to prepare for the next pandemic. Taken together, these recommendations emphasize the need for sustained investment, institutional change, and a more collaborative, integrated approach to the reuse of NTD in public health emergencies.

### Institutionalize Readiness

Across all themes, a central theme emerges: success during the next crisis will not be determined by improvisation or timely responses, but by the foundations we lay today. Being better prepared means building durable technical, organizational, and human capacity. Governments and other stakeholders must work today to formalize roles such as data stewards, establish data sharing agreements, and invest in tools (eg, Fusion Centers [77], Decision Accelerator Labs [78], and simulation exercises) that can be activated quickly when they are needed. Readiness should be seen as a continuous state, supported by ongoing trust building and community involvement. Some specific actions include (1) establishing and institutionalizing data stewards, empowered with formal mandates; (2) creating and funding fusion centers and decision accelerator laboratories to prototype, evaluate, and scale interventions based on nontraditional (and traditional) data; and (3) conducting regular tabletop simulation exercises involving all stakeholders to enhance readiness and identify weak points or challenges.

### Build Data Preparedness

Institutional readiness must be accompanied by data preparedness. Once again, many of the most pressing challenges during the COVID-19 pandemic stemmed from the simple fact that public health representatives were not ready: data were inaccessible and fragmented, and their nature and location were poorly understood. Building data preparedness requires developing standardized approaches to how data are structured, stored, and analyzed—especially for NTDs such as mobility traces or social media signals. Importantly, there is a need to map what data exists and where it resides. Specific actions include (1) developing data inventories and maps to identify nontraditional datasets, assess their accessibility and quality, and ensure metadata are harmonized across sources; (2) launching a multisectoral task force, including epidemic modelers, public health experts, and social scientists to set and maintain data standards for behavioral data integration into models, including protocols for metadata; and (3) create an evidence repository to collate and tag successful (and unsuccessful) uses of NTD during the COVID-19 pandemic to increase data literacy.

### Create a Trusted and Collaborative Ecology

Data do not exist in a vacuum. Its value is realized through relationships among sectors, stakeholders, and, importantly, between those who hold data and those who need it. Trust is the foundation of these relationships and must be deliberately cultivated. This includes establishing clear ethical guidelines for data use but also developing institutional intermediaries such as data stewards and data collaboratives that can manage partnerships and facilitate reuse. An observatory can play a particularly critical role, helping to monitor how NTDs are used in real time, documenting best practices, and ensuring that insights are shared across borders and sectors. Building this ecology will require a cultural shift, from data hoarding to data solidarity.

Specific actions include (1) developing mutual commitment frameworks between private data holders and public entities that may use data to clarify terms of emergency data sharing ahead of crises; (2) creating an observatory for NTD use that tracks deployment, use, and disseminates real-time insights and lessons learned; and (3) encouraging and scaling data collaboratives with clear governance rules and mechanisms for shared infrastructure and trust building across sectors and stakeholders.

Together, these 3 recommendations offer a framework, not a set of independent guidelines or policies. None of these can succeed in isolation, but if pursued together, they can create a virtuous cycle of preparedness, insight, and action.

Crucially, these steps are not only relevant to future pandemics. They can also inform how societies respond to a range of public crises, from climate shocks to humanitarian emergencies. Effective responses to each of these increasingly rely on prepared, tested, informed, and inclusive decision-making.

The COVID-19 pandemic was an indisputable calamity. Nonetheless, it was also a moment of extraordinary innovation, experimentation, and, most importantly, learning. If we absorb its lessons and begin now to build the foundations we lacked, then we may yet emerge from this crisis more resilient and better prepared next time. We may not be able to prevent the next crisis—but we can certainly improve our response and, in so doing, potentially mitigate the scope for another calamity.

## Limitations

This work provides exploratory insights from a purposively sampled community of European epidemic modelers rather than generalizable findings from representative research. The survey was distributed through the ECDC Modeling Hub mailing list and the ESCAPE project networks, reaching modeling teams actively engaged in COVID-19 response efforts. Of the contacts reached, 29 modeling teams responded. All survey results were purely descriptive and reflect only the experiences and perspectives of this specific sample—no inferential statistics or population-level generalizations should be drawn from our findings.

Our recruitment strategy inherently created selection bias toward modelers already familiar with and interested in NTD. Survey participants were recruited through networks focused on epidemic modeling, and workshop participants self-selected to attend an event specifically about NTD integration. This likely resulted in confirmation bias, where reported experiences and needs reflect a community already convinced of the value of NTD sources. Our findings should therefore be interpreted as perspectives from early adopters and advocates of NTD, not as representative of the broader modeling community. The barriers and needs identified may be even more pronounced among modelers not yet engaged with these data sources.

The workshop discussions were captured through structured note-taking by designated members of the organizing team rather than through formal transcription or qualitative coding methods. While this approach is standard for policy-oriented workshops, it introduces subjectivity in how themes were identified and synthesized into our 10 lessons learned. These lessons represent expert recommendations informed by survey data and workshop discussions, not findings that emerged purely from systematic data analysis. All ESCAPE consortium members (a substantial portion of workshop participants and co-authors of this manuscript) reviewed and validated the synthesis, but this does not eliminate the interpretive nature of the process.

Our findings reflect primarily European perspectives and the specific context of the COVID-19 pandemic response. Experiences from other regions may reveal different lessons and suggest alternative recommendations. Similarly, the challenges and opportunities associated with NTD may differ for other types of health emergencies (eg, climate-related health events, humanitarian crises, and endemic disease surveillance).

Nontraditional data sources are not exempt from biases and have their own limitations, including technology adoption bias, noise from self-reporting, unequal representativity among population groups, unequal spatiotemporal coverage, and data ownership and privacy issues. Traditional data exhibit known biases (eg, reporting delays, geographical coverage disparities, and health care accessibility differences) that modelers typically address through Bayesian inference, poststratification, and synthetic data generation. Similar techniques can be applied to NTD, although effective approaches are case specific and data specific.

Despite these limitations, our findings and recommendations are formulated to be applicable beyond the specific context of COVID-19 modeling in Europe. The data types discussed and actions proposed are not disease-specific or geographically constrained, although their implementation may require adaptation to local institutional contexts. This work should be viewed as a starting point for broader discussions on improving data-to-policy translation in public health emergencies, not as definitive guidance derived from representative research.

## Supplementary material

10.2196/85540 Multimedia Appendix 1 Supplementary information with insights on the survey on data readiness and availability.

## Abbreviations

EC: European Commission
EHDS: EU Health Data Space
MNO: mobile network operator
NTD: nontraditional data
