# Supplementary material for: Nontraditional Data in Pandemic Preparedness and Response: Identifying and Addressing First- and Last-Mile Challenges
Source: J Med Internet Res. 2026 Apr 29;28:e85540. doi: 10.2196/85540 (PMC13128049; doi:10.2196/85540)
Supplement: Multimedia Appendix 1 [file jmir-v28-e85540-s001.pdf]

Supplementary material

Survey on data readiness and availability during the COVID-19 pandemic.

Supplementary methods

This study employed a cross-sectional online survey design to assess data availability, use, and unmet needs for the modeling community in epidemiology during the COVID-19 pandemic.

The survey was directed to research groups or individual researchers focused on epidemiological modeling, primarily in the European context. To this end, the survey was disseminated through the ECDC Modeling Hubs mailing list, among modelers in the ESCAPE project and their network, as well as in a high-level expert workshop on the use of non-traditional data during pandemics in Brussels, which included representatives of academia, government agencies, and the private sector. The survey was available between March and October 2024, in the REDCap and LimeSurvey platforms.
The survey had the following specific objectives:

1. To gain a better understanding of the types of data used for modeling during the pandemic, their purpose, and their strengths and limitations, with a particular focus on non-traditional data.
2. To gain a better understanding of the unmet data needs of the modeling community and the underlying causes.

The survey was completely anonymous and consisted of 7 main questions. There were two questions: one regarding “data types used” and another regarding “data types not used but needed.” The options for data types were numerous, and there was free text space for other types not provided. These two questions had several subquestions, allowing us to collect details for each specific data type reported (e.g., details about data, access, and quality, or reasons that prevented the use of data)

The survey was divided into 3 sections.

- The first one aimed to understand the type of research questions and modeling approaches that were used by the research groups/researchers who answered the survey.
- The second section aimed to identify (1) what data was available and used during the pandemic by these research groups; (2) how data was accessed and what problems were encountered in accessing the data; and (3) what quality problems were encountered when using the data.
- The third section aimed to understand what data were needed but not used by these research groups and whether these unmet needs were the result of data not being available or the result of data readiness problems (quality and access issues).

Throughout the survey, the data were categorized into traditional epidemiological data (TD; e.g., tests, hospitalizations, deaths, vaccination, etc.) and non-traditional epidemiological data (NTD), defined as data types not collected with epidemiological purposes and/or relatively new data types (e.g., mobility, contacts, wastewater data, etc.). Thus, in addition to each individual data type, we could have a broader view of traditional vs. non-traditional data.

Supplementary results

In total, 29 research groups/researchers responded to the survey. Mathematical/mechanistic models were the most common method used. The most common research questions were related to the evaluation of control measures, description and short-term prediction of the pandemic, and monitoring of its impact.

The analysis of the survey showed that TD types were the most widely used and available (TD was used by 96% of the respondents and accounts for 67% of the responses)(Fig.3). TD was mainly publicly available or accessed through agreement with governments (83% of responses) (S2 Fig.). However, different types of access and quality problems persist in about 66% and 96% of these cases, respectively (S2 and S3 Fig.). NTD types were commonly used, although less often than TD types (90% of respondents and 33% of responses) (Fig.3). NTD was also often publicly available or accessed through agreements with governments (55% of responses), although

agreements with private institutions and direct access were also very common (S2 Fig.). Hospitalizations and mobility data were the most commonly used TD and NTD types, respectively (used by 82% and 72% of respondents). As for TD, for NTD, different problems with access and quality were also present in around 65% and 96% of cases, respectively (S1 and S3 Fig.).

Regarding the unmet data needs, NTD data types account for more than 72% of the needed but not accessible data (Fig.3). In particular, contact tracing, contacts, and socio-economic data were highly needed but not accessible. Mobility data, although often used, was still highly needed. Regarding the reasons behind these unmet data needs, for both TD and NTD data, unavailability (either total or in adequate time) prevented use in around 60% of the cases (S4 Fig.). Other relevant obstacles for data use were biases (for TD) or temporal and spatial granularity, as well as temporal availability (for NTD).

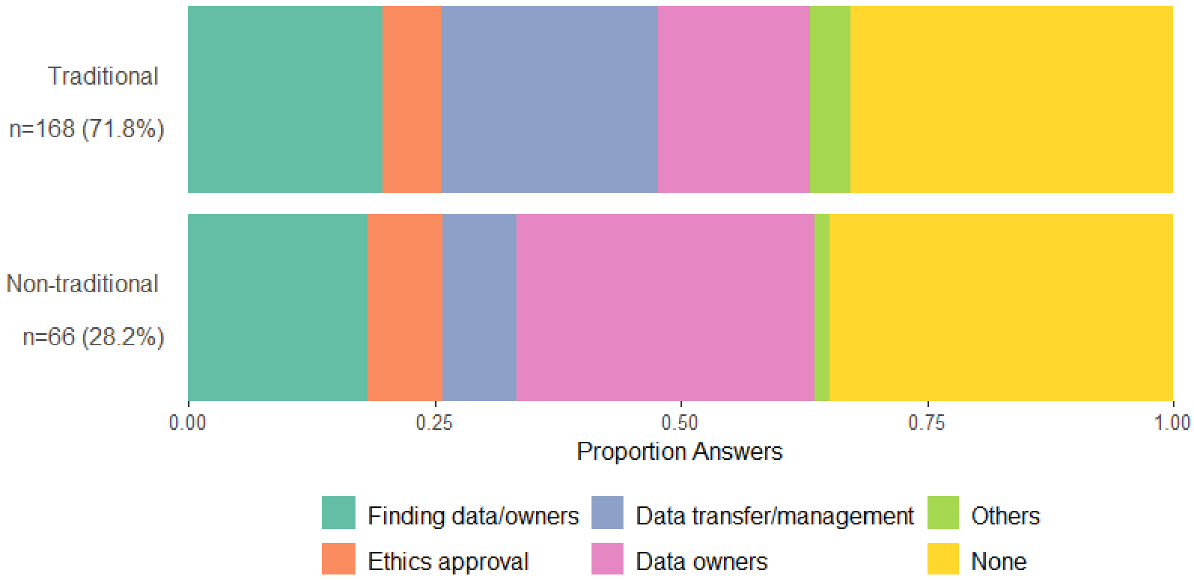

S1 Fig. Problems regarding data access for each of the data types used by respondents, represented by the proportion of answers for each type of data access problem aggregated in TD and NTD. Note that for simplicity, several categories are shown aggregated ("Data owners" aggregates the following categories: data owners reluctant/unwilling to share for privacy, national security, academic competition, and commercial reasons). The total number of answers for TD and NTD, and the percentage of the total number of answers are also shown (note that for each data type, respondents could choose more than one answer).

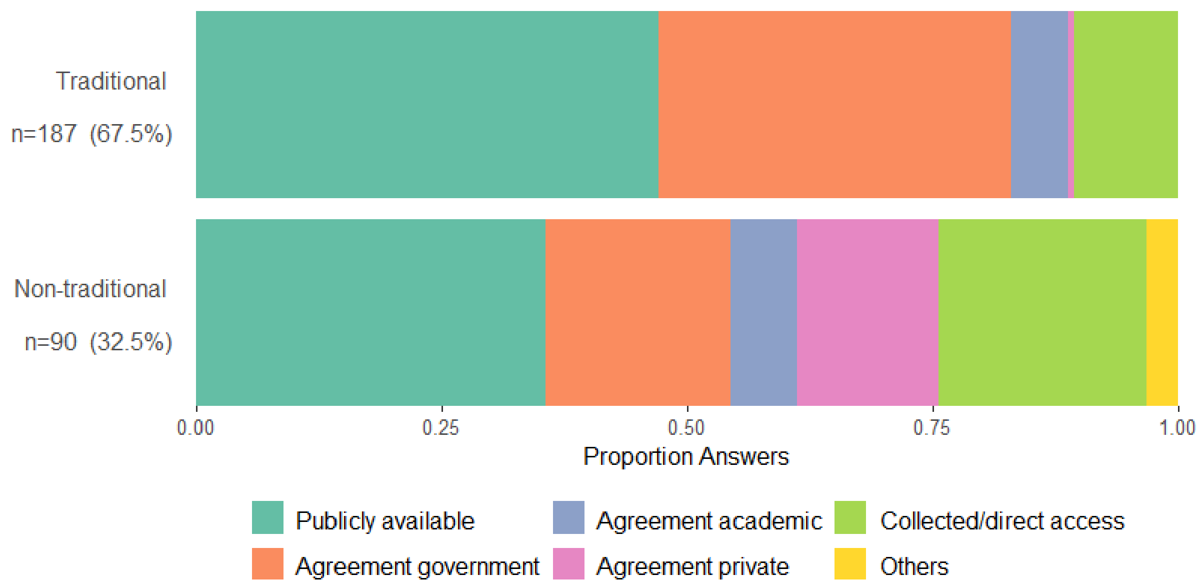

S2 Fig. Type of access for each of the data types used by respondents, represented by the proportion of answers for each type of data access aggregated in TD and NTD. The total number of answers for TD and NTD, and the percentage of the total number of answers are also shown (note that for each data type, respondents could choose more than one answer).

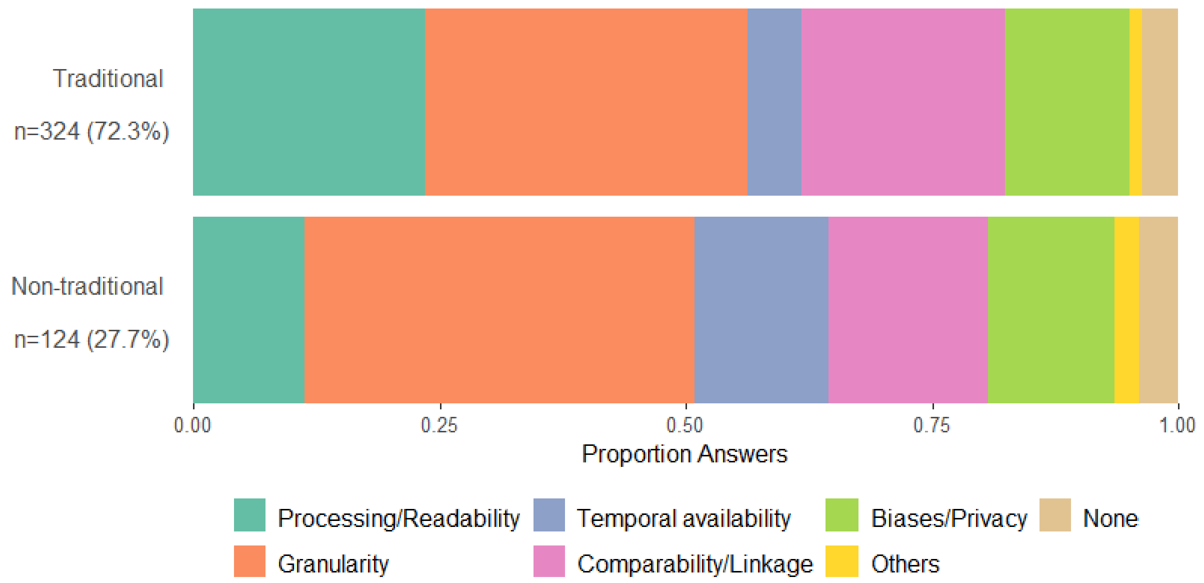

S3 Fig. Problems regarding data quality for each of the data types used by respondents, represented by the proportion of answers for each type of data quality problem aggregated in TD and NTD. Note that for simplicity, several categories are shown aggregated (e.g., "Processing/Readability" aggregates problems with preparation and processing, and with machine readability; "Granularity" aggregates problems with temporal, spatial, and demographical/medical granularity). The total number of answers for TD and NTD, and the percentage of the total number of answers are also shown (note that for each data type, respondents could choose more than one answer).

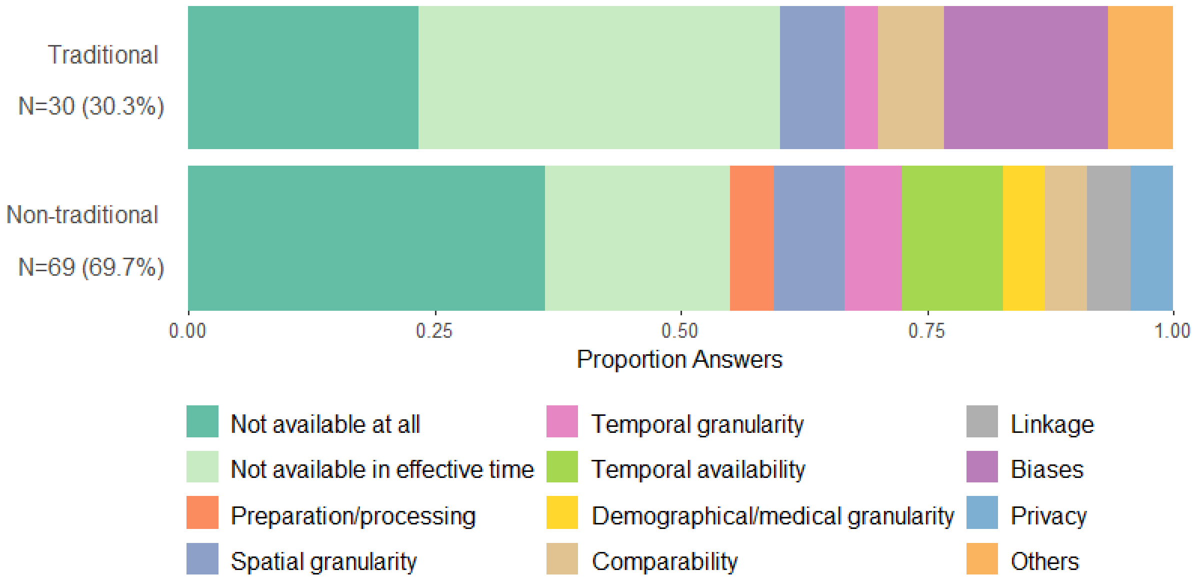

S4 Fig. Reasons that prevented the use of data for each data type not used reported by respondents, represented by the proportion of answers for each of the reasons listed, aggregated in TD and NTD. The total number of answers for TD and NTD, and the percentage of the total number of answers are also shown (note that for each data type, respondents could choose more than one answer).
